# Investigating Maxillary Sinus and Buccal Bone Biometrics via Cone Beam Computed Tomography (CBCT) in an Iranian Sample -

**DOI:** 10.31661/gmj.vi.3935

**Published:** 2025-10-20

**Authors:** Shahriar Shahi, Sahar Ghanbaran, Emad Movahed, AmirMohammad Moharrami, Afsaneh Nekouei, Meysam Mohammadikhah, Shaghayegh Ghadimi

**Affiliations:** ^1^ Department of Endodontics, Faculty of Dentistry, Tabriz University of Medical Sciences, Tabriz, Iran; ^2^ Dental and Periodontal Research Center, Tabriz University of Medical Sciences, Tabriz, Iran; ^3^ Department of Endodontics, School of Dentistry, Shiraz Branch, Islamic Azad University, Shiraz, Iran; ^4^ School of Dentistry, RWTH Aachen University, Aachen, Germany; ^5^ Department of Orthodontics, Faculty of Dentistry, Tehran University of Medical Sciences, Tehran, Iran; ^6^ Dentistry Faculty Shahr_e_kord University of Medical Science, Iran; ^7^ Department of Oral and Maxillofacial Surgery, School of Dentistry, Alborz University of Medical Sciences, Karaj, Alborz, Iran; ^8^ Department of Endodontics, Faculty of Dentistry, Tabriz University of Medical Sciences, Tabriz, Iran

**Keywords:** Maxillary Sinus, CBCT, Buccal Bone Thickness, Root Apex, Anatomical Variations

## Abstract

**Background:**

The maxillary sinus’s close anatomical relationship with posterior teeth roots presents significant challenges in dental and surgical procedures, with variations in sinus morphology influencing clinical outcomes. This study aimed to evaluate maxillary sinus biometrics and buccal bone thickness using Cone Beam Computed Tomography (CBCT) in an Iranian population.

**Materials and Methods:**

A retrospective analysis of 210 CBCT scans was conducted, measuring root apex proximity to the maxillary sinus floor (MSF) and buccal cortical bone thickness.

**Results:**

Findings revealed that 53.06% of right third molar roots protruded into the MSF, while only 2% of left first premolars did. The mesiobuccal root of the third molar had the shortest distance to the MSF (-1.38 ± 0.89 mm), whereas the palatal root of the first premolar was farthest (9.81 ± 3.93 mm). Buccal bone thickness was thinnest at the first premolar (1.21 mm) and thickest at the third molar palatal root (13.23 mm), with significant differences observed among molar roots.

**Conclusion:**

These findings underscore the importance of CBCT in preoperative planning to minimize complications during apical surgery and implant placement, particularly in cases involving posterior maxillary teeth.

## Introduction

While considering the paranasal sinuses, the maxillary sinus is of utmost importance in the fields of dentistry, apical surgery, and oral, maxillofacial, and jaw surgery [1]. Given its anatomical proximity to critical dental structures, understanding its variations is essential for clinical success. Between the roots and the tip of the posterior teeth of the maxilla is where you could find the maxillary sinus floor (MSF). This close relationship often poses clinical challenges, particularly in endodontic and surgical procedures. It is possible for the root apex to enter the sinus cavity in rare instances. When endodontic tools and root-filling materials penetrate the sinus during root canal procedures, the risk of infection and sinusitis increases because the roots are so close to the marginal sinus fibrosus [2][3][4]. Moreover, apical procedures involving the rear teeth of the maxilla can be challenging due to a combination of factors, including the closeness of the MSF and the thickness of the buccal bone, which limits surgical access and the ability to maneuver surgical instruments [5][6].

Anatomical variations further complicate these clinical scenarios. Every person’s maxillary sinus is unique in size and shape. The antral surface of the sinus is elevated or the roots protrude into the sinus in half of the cases because the sinus floor extends between the roots. When this occurs, the sinus floor is much thinner [7]. However, radiographic findings do not always reflect true anatomical relationships. According to histological investigations, the majority of roots that protrude into the sinus on radiographs are really covered by a thin layer of cortical bone. Despite this, perforation remains a significant risk. Perforation of the sinus floor is only seen in 14 to 28 percent of these cases [1].

Recent research has sought to clarify the spatial relationship between the maxillary sinus and posterior teeth roots. There has been a lot of focus lately on the space between the maxillary sinus and the back teeth’s roots. Using CBCT, Razumova et al. (2019) analyzed the connection between 325 patients’ posterior tooth roots and the floor of their maxillary sinus. The shortest distance to the MSF floor was seen in the second molar’s mesiobuccal root, according to the collected data. The comparison of the palatal roots of the first and second molars revealed the most noticeable alteration. Both the large sample size and the use of CBCT technology are strengths of this study [8]. Similarly, to determine the connection between the maxillary sinus and the back teeth of the upper jaw, Kang et al. (2015) used CBCT [9]. Further contributing to this body of research, the maxillary sinus and tooth roots were investigated by Kwak et al. utilizing five separate vertical and three separate horizontal techniques. They found that the root apex was not touching the sinus floor as a result of their vertical relationship assessments. It was common to see the sinus horizontally related to the buccal and palatal roots [10]. Expanding on these findings, the topographical link between the maxillary sinus floor and the roots of the maxillary posterior teeth was investigated by Kalkur et al. (2017) using imaging methods such as optical parametric imaging (OPG) and digital volume tomography (DVT). For 85 individuals, 510 maxillary teeth were examined using OPG and DVT techniques to determine the length of the protrusion over the sinus cavity. They classified the teeth based on how they lined up with the maxillary sinus. The results showed that 85 percent of the roots failed to make contact with the sinus’s cortical edge [11].

Studies focusing on specific populations, such as Iranians, provide additional information. For their descriptive-analytical study, Hekmatian and colleagues (2014) used CBCT to assess, in cross-sectional and panoramic views, the distance between the apices of the upper posterior teeth and the maxillary sinus floor in Iran. Twenty CBCT scans were chosen at random for this study, and their anterior-posterior serially reconstructed cross-sectional slices were evaluated. In both the cross-sectional and panoramic views, the average distance between the tooth root and the floor of the maxillary sinus was 4.17 ± 9.13 mm and 4.22 ± 9.51 mm, respectively. The cross-sectional view for tooth 5 had an average distance of 3.40 ± 7.06 mm, whereas the panoramic view had an average distance of 3.40 ± 7.51 mm. The average distances for tooth 6 in the panoramic view were 2.25 ± 4.91 mm, while in the cross-sectional view they were 2.26 ± 4.73 mm. The average root distances in the panoramic view (1.95 ± 4.30 mm) and the cross-sectional view (1.92 ± 4.01 mm) for tooth 7. Nonetheless, when compared to other research, this one had a tiny sample size [12]. Supporting these findings, Zanganeh (2013) utilized CBCT to examine the correlation between the maxillary sinus and the positioning of the apices of the posterior teeth in the upper jaw [13]. Their research analyzed a total of 110 CBCT pictures. The findings indicate that most of the posterior teeth in the upper jaw did not contact the maxillary sinus. Typically, the buccal and palatal roots of a tooth make contact with the sinus. Moreover, the palatal roots of the first and second molars were observed to invade the sinus more frequently than any other portion of the tooth. This study indicates that the patient’s gender influences the interaction between the maxillary sinus and the roots of the posterior teeth. Further reinforcing these observations, in a descriptive-analytical cross-sectional study, Farhad (2017) employed CBCT to quantify the distance between the roots of the upper and lower jaws (excluding the third molars) and the maxillary sinus and mandibular canal. The study revealed that, for maxillary molars, the mesiobuccal root of the second molar had the lowest distance from the apex to the sinus. Conversely, the distance associated with the mesiobuccal root of the first molar was the most significant. Moreover, girls had a significantly greater average distance from the apices of the palatal roots of the left side molars compared to boys. Different age cohorts exhibited statistically significant variations in the average distances from the apices of the distobuccal, mesiobuccal, and palatal roots of the first maxillary molar to the sinus [14].

Given these complexities, clinical awareness is crucial. The proximity of the maxillary sinus to the back teeth’s tips is a common concern among patients undergoing procedures in this area. A comprehensive understanding of the region’s anatomy can help reduce the chances of therapeutic intervention complications. However, gaps remain in the literature regarding the Iranian population. Due to a lack of research including large enough samples from the Iranian population, this study aims to use CBCT to evaluate buccal bone thickness and biometrics of the maxillary sinus.

## Materials and Methods

### Study Design

This descriptive-analytical retrospective study was based on radiographic data obtained from CBCT scans between February 2018 and December 2020 in dental clicnics of Tabriz University of Medical Sciences. This study utilized archived CBCT images from the radiology department of the Tabriz University of Medical Sciences Dental School. All ethical principles were adhered to, and patient information was kept confidential. The study was approved by the Ethics Committee of Tabriz University of Medical Sciences under approval number IR.TBZMED.VCR.REC.1399.649. The sample size was calculated following the method described by Kang [9], which considered the mean distance between the apices of maxillary teeth and the maxillary sinus floor (Group 1: 0.75 ± 0.21 mm; Group 2: 6.61 ± 4.46 mm) with a significance level (α) of 0.05 and 80% statistical power. A minimum of 208 participants was required to ensure statistical robustness.

### Inclusion and Exclusion Criteria

The study included CBCT images that met specific inclusion criteria: clear visualization of at least one maxillary sinus region, fully formed root apices (closed apices), and maxillary molars or premolars without periapical bone destruction. Exclusion criteria comprised CBCT scans with artifacts or poor diagnostic quality, teeth exhibiting periapical lesions, a history of orthodontic treatment, or patients with prior trauma, sinus pathology, or intraosseous lesions.

### Participants and Imaging Protocol

A total of 210 participants who underwent CBCT imaging for diagnostic or therapeutic purposes between 2018 and 2020 were included. Imaging was performed using a NewTom VGi CBCT scanner (NewTom, Verona, Italy) with a full 360° rotation and a flat-panel detector. The exposure parameters were set at 110 kVp and a variable current of 1-20 mA. Each scan had a duration of 18 seconds, a field of view (FOV) of 15 × 15 cm, and an exposure time of 3.6 seconds. Cross-sectional slices were reconstructed at 0.3 mm thickness for analysis.

### Image Analysis

All measurements were conducted using NNT Viewer software (NewTom) under the supervision of a board-certified oral and maxillofacial radiologist. The distance from the buccal cortical bone surface to the root apex was measured for all roots (single-, double-, or triple-canal) on both the right and left sides. Additionally, the distance from the root apex to the maxillary sinus floor was measured bilaterally with a precision of 0.1 mm. Images were displayed on a high-resolution monitor (1920 × 1536 pixels) with a 14-bit pixel density and a pixel size of 127 × 127 µm² to ensure accurate assessment.

### Statistical Analysis

Data were analyzed using SPSS version 17 (IBM Corp., Armonk, NY, USA). Continuous variables were reported as mean ± standard deviation (SD), while categorical variables were presented as frequencies (%). Independent t-tests and analysis of variance (ANOVA) were used for group comparisons, with statistical significance set at p < .05.

## Results

**Table:** Table1. Distribution of Root Apex Position Relative to the Maxillary Sinus Floor (MSF) in Different Tooth Groups (G1: Protrusion into MSF, G2: Contact with MSF, G3: Distant from MSF)

		Right			Left	
Tooth	G1	G2	G3	G1	G2	G3
Third Molar	26 (53.06%)	20 (40.82%)	3 (6.12%)	26 (50%)	21 (40.38%)	5 (9.62%)
Second Molar	26 (36.11%)	35 (48.61%)	11 (15.28%)	25 (34.72%)	36 (50%)	11 (15.28%)
First Molar	25 (12.89%)	95 (48.97%)	74 (38.14%)	24 (12.77%)	92 (48.94%)	72 (38.3%)
Second Premolar	5 (2.43%)	80 (38.83%)	121 (58.74%)	5 (2.4%)	82 (39.42%)	121 (58.17%)
First Premolar	4 (2.03%)	48 (24.37%)	145 (73.6%)	4 (2%)	55 (27.5%)	151 (75.5%)

**Table:** Table2. Comparison of the Distance between the Apex of the Root and the Floor of the Maxillary Sinus in Different Directions and On the Right and Left Sides of the Molar Teeth (G1: Protrusion into MSF, G2: Contact with MSF)

	Side	Group	Distobuccal	palatal	mesiobuccal	P-value
	Right	G1	-1.79(0.91)	-1.43(0.98)	-1.62(0.83)	<.05
Third Molar		G3	2.24(1.12)	1.98(0.95)	2.19(0.93)	<.05
	Left	G1	-1.61(0.84)	-1.38(0.89)	-1.54(0.88)	<.05
		G3	2.19(0.97)	1.95(0.96)	2.12(1.08)	<.05
	Right	G1	-1.49(0.98)	-0.95(0.85)	-1.01(0.81)	<.05
Second Molar		G3	3.82(1.9)	4.11(1.94)	3.62(1.88)	<.05
	Left	G1	-1.42(0.93)	-0.91(0.89)	-0.86(0.79)	<.05
		G3	3.75(1.87)	4.05(1.66)	3.47(1.69)	<.05
	Right	G1	-1.1(0.85)	-0.92(0.69)	1.05(0.87)	0.234
First Molar		G3	4.6(2.31)	4.72(2.3)	4.53(2.21)	0.297
	Left	G1	-1.07(0.82)	-0.9(0.65)	1.01(0.81)	0.316
		G3	4.51(2.11)	4.63(2.1)	4.47(2.18)	0.289

**Table:** Table3. Comparing the Distance between the Apex of the Root and the Floor of the Maxillary Sinus in Different Directions and On the Right and Left Sides of the Premolar Teeth

			Palatal	Buccal	
**First Premolar**	Right	G1	-0.71(0.28)	- (- )	P-value
		G3	9.8(3.85)	9.7(4.12)	0.462
	Left	G1	-0.62(0.31)	-0.41(0.28)	0.133
		G3	9.81(3.93)	9.62(4.34)	0.498
**Second Premolar**	Right	G1	-0.89(0.85)	-1.2(0.96)	0.153
		G3	7.95(4.37)	7.46(4.28)	0.295
	Left	G1	-0.82(0.91)	-1(0.85)	0.097
		G3	7.82(4.71)	7.33(4.2)	0.357

**Table:** Table4. Comparison of Buccal Cortical Thickness in Three Types of Molars On the Right and Left Sides

		Palatal	Mesiobuccal	Distobuccal	P-value
Third Molar	Right	13.23 (1.09)	5.29(1.28)	6.57(1.14)	0.001
	Left	13.07 (2.64)	5.18(1.39)	6.53(1.57)	0.001
Second Molar	Right	12.1 (1.67)	4.28(1.09)	5.18(1.11)	0.001
	Left	12.11 (1.36)	4.26(1.14)	5.23(1.04)	0.001
First Molar	Right	12.19 (1.63)	3.18(0.98)	3.01(1.22)	0.001
	Left	12.13 (1.29)	3.06(1.04)	3.08(155)	0.001

**Table:** Table5. Comparison of Buccal Cortical Thickness in Right and Left Premolars

		Palatal	Buccal	P-value
Second Premolar	Right	6.1(1.39)	2.51(0.83)	0.001
	Left	6.13(1.25)	2.49(1.19)	0.001
First Premolar	Right	5.09(2.19)	1.28(0.84)	0.001
	Left	4.52(2.32)	1.21(0.94)	0.001

The sample consisted of 210 CBCT images, with a gender distribution of 48.1% male and 51.9% female. The number of teeth observed varied by type and side: on the right side, there were 197 first premolars, 206 second premolars, 194 first molars, 72 second molars, and 49 third molars; on the left side, there were 200 first premolars, 208 second premolars, 188 first molars, 72 second molars, and 52 third molars.

### Root Apex Position Relative to the Maxillary Sinus Floor (MSF)

The position of root apices relative to the MSF varied significantly across tooth types (Table 1). Among third molars, 6.12% of right-sided and 9.62% of left-sided roots were distant from the MSF. For second molars, 15.28% of roots on both sides were distant from the MSF. A higher proportion of first molar roots were distant from the MSF (right: 38.14%; left: 38.3%). Second premolars exhibited the highest distant positioning (right: 58.74%; left: 58.17%), followed by first premolars (right: 73.6%; left: 75.5%).

### Distance Between Root Apex and MSF

Among molars, the mesiobuccal root of the third molar (Group 1) had the shortest distance to the MSF (right: −1.62 ± 0.83 mm; left: −1.54 ± 0.88 mm), while the mesiobuccal root of the first molar (Group 3) had the longest distance (right: 4.53 ± 2.21 mm; left: 4.47 ± 2.18 mm). Significant differences were observed among the roots of the third and second molars (P < .05), but not among first molar roots (P > .05; Table 2).

For premolars, the second premolar (Group 1) had the smallest mean distance to the MSF (right: −0.89 ± 0.85 mm), while the first premolar (Group 3) had the largest (right: 9.80 ± 3.85 mm). No significant differences were found between the roots of first and second premolars (P > .05; Table 3).

### Buccal Cortical Bone Thickness

In molars, the palatal roots exhibited the greatest buccal cortical thickness on both sides (third molar right: 13.23 ± 1.09 mm; left: 13.07 ± 2.64 mm; second molar right: 12.10 ± 1.67 mm; left: 12.11 ± 1.36 mm; first molar right: 12.19 ± 1.63 mm; left: 12.13 ± 1.29 mm), with significant differences among roots (p = .001; Table 4).

Similarly, in premolars, the palatal roots had significantly thicker buccal cortical bone than buccal roots (second premolar right: 6.10 ± 1.39 mm vs. 2.51 ± 0.83 mm; left: 6.13 ± 1.25 mm vs. 2.49 ± 1.19 mm; first premolar right: 5.09 ± 2.19 mm vs. 1.28 ± 0.84 mm; left: 4.52 ± 2.32 mm vs. 1.21 ± 0.94 mm; all p = .001; Table 5).

## Discussion

The primary objective of this study was to examine maxillary sinus biometrics and buccal bone thickness using CBCT. The findings revealed significant variations in root proximity to the maxillary sinus floor (MSF) across different tooth types. The number of root apices extending into the MSF was highest in third molars and lowest in first premolars. Notably, the palatal root of the first premolar exhibited the longest distance from the root tip to the MSF, whereas the mesiobuccal root of the third molar had the shortest. Furthermore, significant differences were observed among the three roots of the third molar, with the mesiobuccal roots on both sides showing the closest proximity to the sinus. A similar pattern was seen in second molars, where the mesiobuccal roots again demonstrated the shortest distance. In contrast, the first molar group showed no statistically significant variation in root spacing. While the first and second premolars displayed no marked differences in root length, the palatal roots were slightly longer than the buccal roots. Regarding buccal bone thickness, the pulpal root of the first premolar had the shortest distance to the buccal cortical bone, while the palatal root of the third molar had the greatest (13.23 mm vs. 1.21 mm).

These anatomical relationships hold critical implications for clinical practice. When operating on the posterior maxilla, dentists must remain mindful of the close proximity between the maxillary sinus and dental roots. Early attempts to classify these relationships were limited. Freisfeld et al. proposed a classification system for dental bridges and sinus connections but focused solely on the first molar, neglecting sinus topography and root positioning [15]. A more comprehensive approach was later introduced by Kwak et al., who developed a CT-based classification system [10]. Despite numerous studies on root protrusion into the sinus, inconsistent criteria have led to variability in findings. For instance, a root may appear to intrude into the sinus in one radiographic view but not in another. To address this, our study, like Kang et al.’s, classified roots as protruding only if they appeared to contact the sinus in all dimensions [9]. Consistent with prior research, we found that group one roots (those intruding into the MSF) were more prevalent in posterior teeth.

Supporting these observations, Kang et al. reported that projecting roots were more common in posterior teeth, with frequencies of 1.5% in first premolars, 14.8% in second premolars, 40.5% in first molars, and 44.7% in second molars [9]. Our results align closely with these findings, likely due to the shared use of CBCT for evaluation. However, discrepancies exist with other studies. Kalkur et al. found that 85% of roots did not contact the MSF, but their reliance on panoramic radiography, a less precise method, may explain this divergence [10]. Similarly, DehghaniTafti et al. reported that only 13.5% of first molars fell into group one, while 65.2% of first premolars were in group three (root apex distant from the sinus) [16]. These differences may stem from methodological limitations, as their study lacked advanced imaging validation.

Notably, no premolar buccal roots in our study were classified as group one, mirroring Kang et al.’s findings [9]. However, Von et al. [17] reported minor protrusion rates for premolar roots (2.5%-7.7% for buccal roots and 8.7%-13.6% for palatal roots). These slight variations likely reflect demographic differences across study populations.

Key findings from our study include: The palatal root of the first premolar was farthest from the MSF, while the mesiobuccal root of the third molar was closest; Significant differences existed among third and second molar roots, with mesiobuccal roots consistently closest to the sinus; No significant differences were noted in first molar root distances.

These results corroborate prior research. Kang et al. found that 35.8% of second molar mesiobuccal roots intruded into the MSF [9], while Razumova et al. identified the shortest MSF distance in these roots [8]. In a postmortem study of an Iranian population, Poorebrahim et al. confirmed that the mesiobuccal root of the second molar was closest to the MSF [18]. Similarly, Farhad et al. reported the shortest apex-to-sinus distance in second molar mesiobuccal roots [14]. Collectively, these studies underscore the reliability of CBCT in assessing root-sinus relationships.

Buccal bone thickness is another critical factor in treatment planning. Apical procedures often require buccal access, making bone thickness a key consideration. Our study found the thinnest buccal bone at the first premolar (1.21 mm) and the thickest at the third molar palatal root (13.23 mm).

These findings align with existing literature. Mogharrabi et al. reported >1 mm buccal bone thickness at second premolars, while first premolars averaged ~1 mm [19]. Similarly, Tsigarida et al. noted increasing buccal bone thickness from anterior to posterior regions [20], and Jin et al. observed <2 mm thickness near canines and first premolars versus >2 mm at second premolars [21].

### Limitations and Recommendations

The study’s single-center design and limited age/cultural diversity may restrict generalizability. Convenience sampling could inadvertently skew results. Multi-center studies are needed to enhance validity.

## Conclusion

The root apices situated within the MSF were found in 53.06% of the right third molars and in 2% of the left first premolars. The average distance from the root apex to the floor of the maxillary sinus was 9.81 ± 3.93 mm, which was the longest for the palatal root of the first premolar. In comparison, the third molar’s mesiobuccal root had the lowest distance, measuring -1.38 ± 0.89 mm. The distance from the root apices to the cortical bone plate was least for the buccal root of the first premolar and highest for the palatal root of the third molar.

## Conflict of Interest

The authors have no conflicts of interest to declare.
